# Enterovirus-related diarrhoea in Guangdong, China: clinical features and implications in hand, foot and mouth disease and herpangina

**DOI:** 10.1186/s12879-016-1463-9

**Published:** 2016-03-16

**Authors:** Hong-Tao Zhou, Hai-Su Yi, Yong-Hui Guo, Yu-Xian Pan, Shao-Hua Tao, Bin Wang, Man-Jun Chen, Mei Yang, Nan Yu

**Affiliations:** Laboratory of Emerging Infectious Diseases and Division of Laboratory Medicine, Zhujiang Hospital, Southern Medical University, No. 253 Gong Ye Da Dao Zhong, Guangzhou, 510282 China; Department of Pediatrics, Zhujiang Hospital, Southern Medical University, Guangdong, 510282 China; Department of Clinical Laboratory, Hainan Provincial People’s Hospital, No. 19 Xiuhua Road,, Haikou, 570311 China; Department of Infectious Diseases, Zhujiang Hospital, Southern Medical University, Guangdong, 510282 China

**Keywords:** Enterovirus, Diarrhoea, Hand, Foot and mouth disease, Herpangina, Clinical features

## Abstract

**Background:**

A series of complications caused by enteroviruses, including meningitis, encephalitis, acute flaccid paralysis, acute cardiopulmonary failure, respiratory infection, and myocardial injury have been reported in hand, foot and mouth disease/herpangina (HFMD/HA). However, the complication of diarrhoea caused by enteroviruses has been neglected, and a summary of its clinical features and impact on HFMD/HA is unavailable.

**Methods:**

We included inpatients with HFMD/HA admitted to the Paediatric Department of Zhujiang Hospital during 2009–2012. We summarised and compared clinical data for cases with and without diarrhoea, and determined enterovirus serotypes by reverse transcriptase polymerase chain reaction and genotyping based on a partial-length fragment of viral protein 1 or the 5’-untranslated region.

**Results:**

There were 804 inpatients with HFMD/HA and 28 (3.5 %) presented with diarrhoea. Gastrointestinal symptoms were mild in most cases of diarrhoea (82.1 %), with high prevalence of no dehydration (82.1 %), short duration of diarrhoea (78.6 %) and watery stools (75.0 %). The prevalence of multi-organ dysfunction syndrome (10.7 vs 0.40 %) (*p* = 0.001), hepatic injury (14.3 vs 3.4 %) (*p* = 0.019), myocardial injury (21.4 vs 6.1 %) (*p* = 0.002) and convulsion (21.4 vs 7.2 %) (*p* = 0.016) was significantly higher in the diarrhoea than no diarrhoea group. There was no significant difference between the two groups regarding prevalence of death, altered consciousness, paralysis, central nervous system involvement, or acute respiratory infection.

**Conclusions:**

Most patients with diarrhoea caused by enteroviruses circulating in Guangdong Province in 2009–2012 had mild or moderate gastrointestinal symptoms. Although enterovirus-related diarrhoea caused additional multi-organ dysfunction syndrome, hepatic injury and myocardial injury in children with HFMD/HA, timely intervention efficiently reduced disease severity and improved outcome.

## Background

Enteroviruses (EVs) are known to cause hand, foot and mouth disease/herpangina (HFMD/HA), with numerous epidemics worldwide, including China [1]. Several complications have been reported in HFMD/HA, including meningitis, encephalitis, acute flaccid paralysis, acute cardiopulmonary failure, respiratory infection, myocardial injury, and diarrhoea [2, 3]. Among these complications in HFMD/HA, diarrhoea is less prominent because of its low prevalence and generally self-limiting nature. In China, diarrhoea has generally been neglected or only mentioned in most studies of HFMD/HA. Moreover, EV detection is often not included in pathogen surveillance of diarrhoea; thus, little information on the epidemiology and clinical features of enterovirus-related diarrhoea is available in China. Diarrhoea can cause various degrees of illness, ranging from self-limited changes in stool consistency to life-threatening complications and even death. Diarrhoea deserves attention because it is the third leading cause of death in young children, claiming millions of lives annually worldwide [4].

Considering the global presence of HFMD/HA, there is an urgent need to elucidate the clinical characteristics of enterovirus-related diarrhoea and its impact on HFMD/HA. Therefore, the purpose of this study was to describe the clinical features and implications of enterovirus-related diarrhoea in children with HFMD/HA admitted to Zhujiang Hospital during 2009–2012.

## Methods

### Study population and data collection

All cases were inpatients admitted to the Paediatric Department of Zhujiang Hospital between January 2009 and December 2012. Zhujiang Hospital is an officially designated tertiary hospital for treatment of HFMD in Guangzhou City (capital of Guangdong Province, China) and its neighbouring districts. Anonymous processing of patient information ensured the privacy of patients, and the study was approved by the Ethics Committee of Zhujiang Hospital (Ratification No: ZJYY-2013-YXJYZX-001). Signed informed consent was obtained from parents or guardians. Cases were included if they met all of the inclusion criteria and none of the exclusion criteria. Inclusion criteria were: paediatric inpatients with eruptions or fever; and real-time fluorescence reverse transcriptase polymerase chain reaction (RT-PCR) evidence of positive enterovirus. Exclusion criteria were: medical history of neurological disease, congenital heart disease or immune deficiency; or evidence of infection by rotavirus, norovirus, *Shigella*, *Campylobacter* or *Salmonella* species. Clinical data for each case were retrospectively reviewed and extracted from medical records, and patients were assigned to the diarrhoea or no diarrhoea group, depending on whether they presented with diarrhoea. Clinical features were compared between the two groups to explore the implications of diarrhoea in HFMD/HA.

### Stool sample collection and testing

Stool specimens from each case were collected for extraction of RNA and stored at −80 °C. Viral RNAs were extracted from 140-μl stool samples using the QIAamp Viral RNA Mini Kit system (Qiagen, Hilden, Germany). Extracted RNAs were used for real-time fluorescence RT-PCR and/or the amplification of partial-length fragments of enterovirus viral protein (VP)1/5’-untranslated region (UTR). Serotypes of enterovirus 71 (EV71) and coxsackievirus 16 (CA16) were jointly determined by enteroviruses kit, EV71 kit, and CA16 kit (Shanghai ZJ Bio-Tech Co. Ltd., China). The other serotypes were determined by sequencing partial-length fragments of VP1/5’-UTR, as described previously [5, 6]. The enterovirus serotype was ascertained by performing BLAST analysis along with EV sequences in GenBank. Each patient with diarrhoea received routine stool examinations, and samples were tested for bacterial cultures of *Shigella*, *Campylobacter* and *Salmonella* species. Routine stool examinations include assessment of colour, consistency, parasite ova, white blood cells, red blood cells, occult blood, transferrin, and rotavirus and norovirus antigens.

### Clinical definitions

Diarrhoea was defined as a change in stool consistency, or defecation ≥3 times/day. The severity of diarrhoea was assessed by a scoring system based on vomiting, frequency and duration of diarrhoea, electrolyte disturbance, dehydration status, and fever. A total score >11 was classified as serious diarrhoea, and ≤11 as mild diarrhoea [7]. Central nervous system (CNS) involvement referred to the presence of two or more of the following: vomiting, irritability, myoclonic jerk, convulsion, changed muscular tension, decreased tendon reflex, headache, and neck stiffness. Patients who also had altered consciousness, paralysis, or CNS lesions on neuroimaging were considered to have severe CNS involvement. Acute respiratory infection (ARI) was diagnosed by the presence of rhinorrhoea and/or cough, and patients with pneumonia or bronchopneumonia were judged to have severe (ARI). Myocardial injury was diagnosed based on laboratory evidence of elevated cTnI, or elevated CK-MB plus CK-MB/CK > 0.25. cTnI > 0.04 μg/l, ratio of CK-MB/CK > 0.25 and CK-MB > 44 U/L (2 m–36 m), CK-MB > 37 U/L (37 m–72 m), or CK-MB > 31 U/L (>73 m) were considered elevated. Cases with alanine aminotransferase >40 U/l were considered to have hepatic injury.

### Statistical analysis

Statistical analysis was performed by SPSS version 13.0 (SPSS, Chicago, IL, USA). Differences between two groups of continuous variables were analysed by the independent samples *t*-test, whereas those of categorical variables were analysed by the *χ*^2^ test. All *p* values were two-sided, and *p* < 0.05 was considered statistically significant.

## Results

**Fig. 1:**
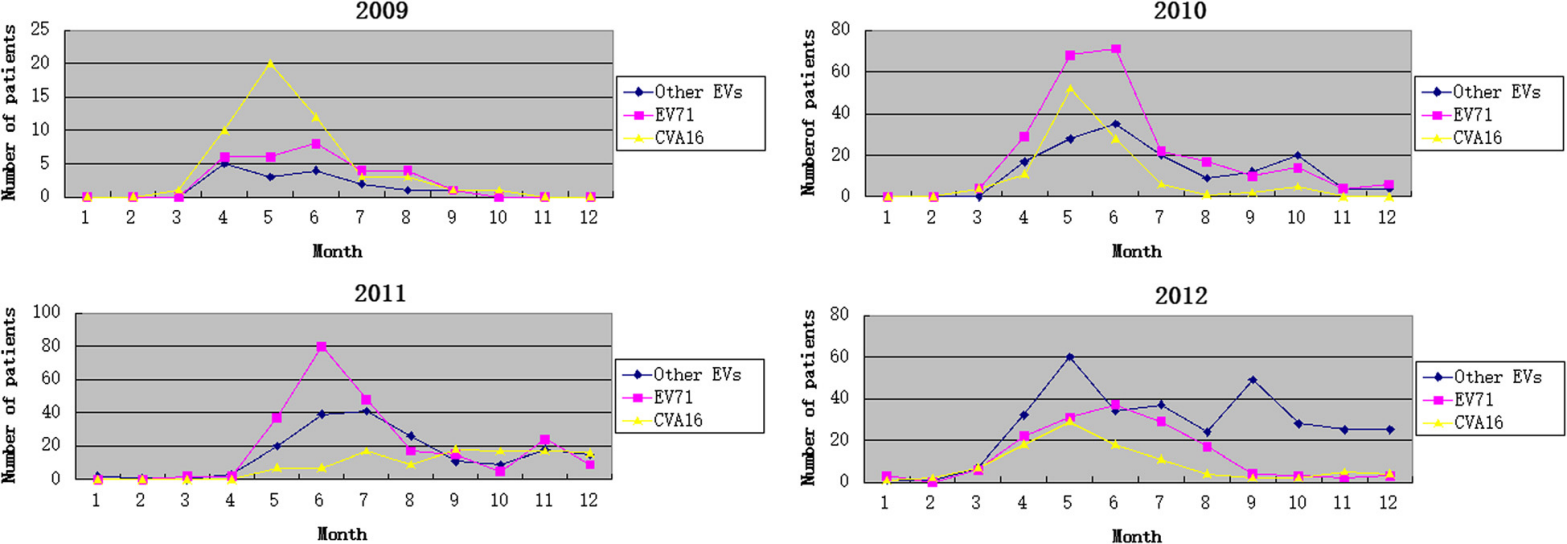
Distribution of HFMD/HA cases caused by EV71, CVA16 and other enteroviruses admitted during 2009–2012

**Fig. 2:**
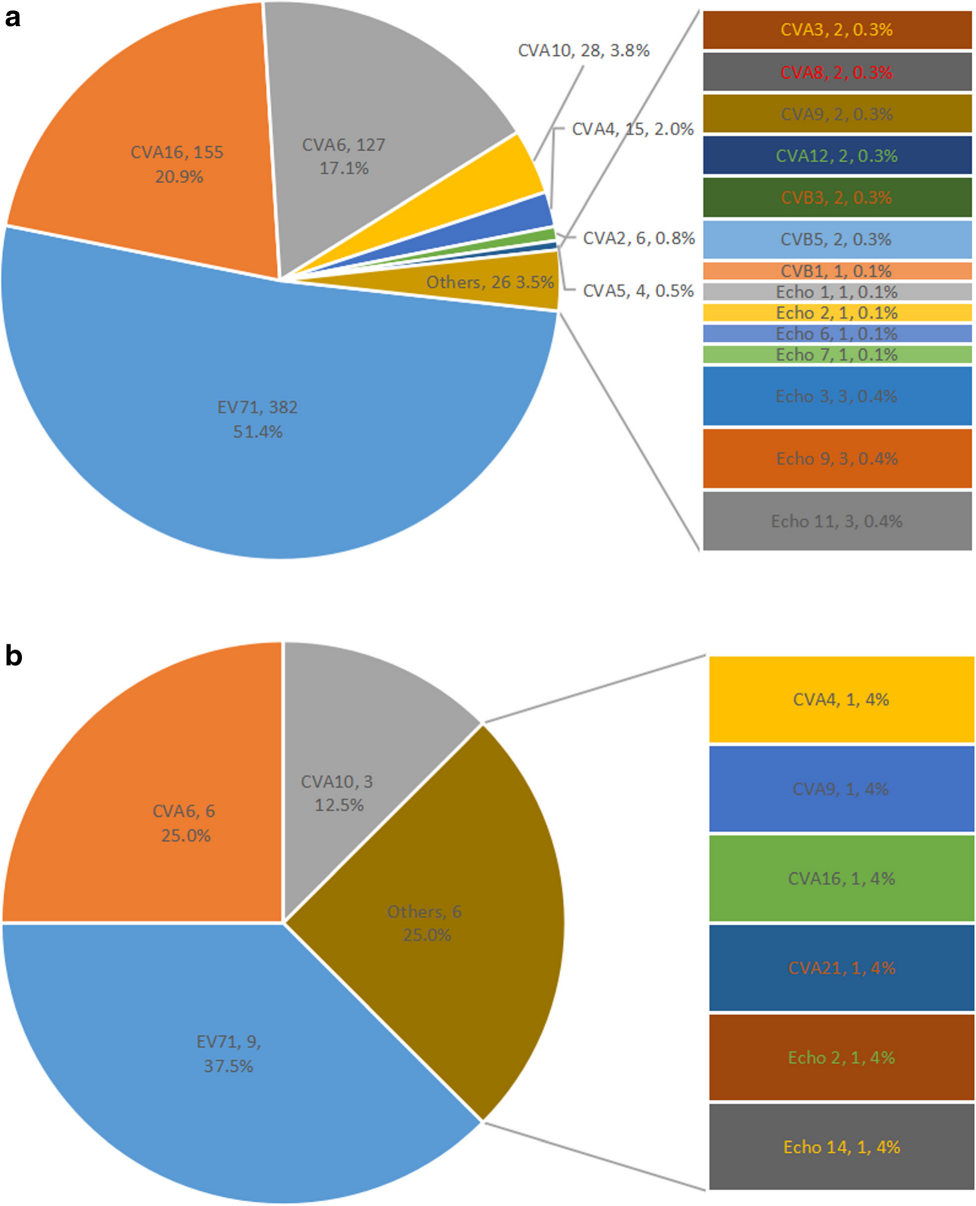
**a** Constituent ratios of EV serotypes in group without diarrhoea. **b** Constituent ratios of EV serotypes in group with diarrhoea

A total of 1778 EV-related HFMD/HA patients were admitted to the Paediatric Department of Zhujiang Hospital during 2009–2012. There were 872 inpatients and 896 outpatients. Most patients with HFMD/HA were admitted in April to July, with a peak between May and June (Fig. 1). There was a shift in the dominant enterovirus serotype during 2009–2012: CA16 in 2009, EV71 in 2010–2011, and other EV serotypes in 2012 (Fig. 1). There was a low prevalence of enterovirus-related diarrhoea and varied prevalence of different EV serotypes. Of the 872 inpatients, 804 were eligible for inclusion in this study, and 28 presented with diarrhoea (3.5 %). We identified enterovirus serotypes in 767 cases, and the detailed distribution of the serotypes in the no diarrhoea and diarrhoea groups is shown in Fig. 2a and b, respectively. Diarrhoea prevalences of different EV serotypes varied considerably: Echo 14 (100 %), CVA21 (100 %), Echo 2 (50.0 %), CVA9 (33.3 %), CVA10 (9.7 %), CVA4 (6.3 %), CVA6 (4.5 %), EV71 (2.3 %) and CVA16 (0.6 %).

### Severity and gastrointestinal symptoms of enterovirus-related diarrhoea

Based on the scoring system, the 28 cases of diarrhoea had a score of 4–17, and 23 cases (82.1 %) scored ≤11. Table 1 shows that most patients with enterovirus-related diarrhoea had mild gastrointestinal symptoms, as characterised by high prevalence of no dehydration (82.1 %), short duration of diarrhoea (78.6 %) and watery stools (75.0 %). Two patient with diarrhoea caused by EV71 died: one died from cardiopulmonary failure, and the other from multi-organ dysfunction syndrome (MODS).

**Table 1:** Clinical presentations of 28 enterovirus-related HFMD/HA inpatients with diarrhoea

Characters	*N* (%)
Age (months)	28.7 ± 17.7
Duration of diarrhoea (days)	
1–4	22 (78.6)
5	0 (0)
≥6	6 (21.4)
Maximum no. diarrhoeal stools/24 h	
≤3	10 (35.7)
4–5	8 (28.6)
≥6	10 (35.7)
Vomiting	11 (39.3)
Duration of vomiting (days)	
0	17 (60.7)
1	7 (25.0)
2	1 (3.6)
≥3	3 (10.7)
Maximum no. vomiting episodes/24 h	
0	17(60.7)
1	0 (0.0)
2–4	8 (28.6)
≥5	3 (10.7)
Maximum body temperature (°C)	
<37.1	1 (3.6)
37.1–38.4	2 (7.1)
38.5–38.9	9 (32.1)
≥39	16 (57.1)
Dehydration	
None	23 (82.1)
Mild	1 (3.6)
Severe	4 (14.3)
Stool consistency	
Watery	21 (75.0)
Mushy	6 (21.4)
Mucous	1 (3.6)
Occult blood in stool	4 (14.3)
Mild diarrhoea (score ≤ 11)	23 (82.1)
Severe diarrhoea (score > 11)	5 (17.9)

### Effect of enterovirus-related diarrhoea on outcomes and severity of HFMD/HA

There were no significant differences between the diarrhoea and no diarrhoea groups for duration of hospitalisation and prevalence of death, altered consciousness, paralysis, CNS involvement, severe CNS involvement, ARI and severe ARI (Table 2). Compared with the no diarrhoea group, the diarrhoea group had a significantly higher prevalence of MODS (10.7 vs 0.40 %) (*p* = 0.001), hepatic injury (14.3 vs 3.4 %) (*p* = 0.019), myocardial injury (21.4 vs 6.1 %) (*p* = 0.002) and convulsion (21.4 vs 7.2 %) (*p* = 0.016).

**Table 2:** Comparisons of clinical features between patients with and without diarrhoea

Characteristics	Diarrhoea (%) (*n* = 28)	No diarrhoea (%) (*n* = 776)	*P*
Demographic			
Diarrhoea (months)	18.3 ± 21.2	29.1 ± 17.6	0.015
Male-to-female ratio	1.55:1	2.00:1	0.506
Fever ≥ 39 °C	16 (57.1)	459 (59.1)	0.832
Fever duration ≥ 3 days	20 (71.4)	446 (57.5)	0.142
Elevated ALT	4 (14.3)	23/675 (3.4)	0.019
Elevated myocardial enzyme	6 (21.4)	30/703 (6.1)	0.002
Convulsion	6 (21.4)	56 (7.2)	0.016
Altered consciousness	4 (14.3)	65 (8.5)	0.291
Severe CNS involvement	4 (14.3)	86 (11.1)	0.543
CNS involvement	12 (42.9)	233 (30.0)	0.147
Severe ARI	5 (17.9)	99 (12.8)	0.393
ARI	11 (39.3)	244 (31.4)	0.410
MODS	3 (10.7)	3 (0.40)	0.001
Paralysis	0 (0.0)	28 (3.6)	0.619
Mortality	2 (7.1)	12 (1.5)	0.082
Duration of hospitalization (days)	6.0 ± 2.7	6.3 ± 4.2	0.792

Note. *ALT* alanine aminotransferase, *ARI* acute respiratory infection, *CNS* central nervous system, *MODS* multi-organ dysfunction syndrome

## Discussion

Our study demonstrated that most cases of enterovirus-related diarrhoea (82.1 %) were mild without severe gastrointestinal symptoms. Among the 28 cases of diarrhoea, there was a low prevalence of severe dehydration (14.3 %), frequent episodes of diarrhoea (≥6/day) (35.7 %), long duration of diarrhoea (≥6 days) (21.4 %), visible bloody stool (0 %), frequent vomiting (≥5 episodes/day) (10.7 %) and long duration of vomiting (≥3 days) (10.7 %). Our study also showed a low prevalence (3.5 %) of diarrhoea caused by enteroviruses circulating in Guangdong during 2009–2012. Although enteroviruses are not well known for causing diarrhoea, some studies have shown them to be causative agents [8–12]. Some other members of the Picornaviridae, such as Aichi virus, parechovirus, and bocavirus, have been recently associated with diarrhoea; however, only with a small number of cases. Therefore, there was only a small probability that any of the cases of diarrhoea in the present study were caused by co-infection with these viruses and enteroviruses. Rotavirus, norovirus, and *Shigella* have been reported as major causative pathogens of diarrhoea in Guangdong Province and its neighbouring districts [13–16]. Although we did not perform comprehensive tests to exclude all possible cofactors of diarrhoea, it is reasonable to suggest that enteroviruses were responsible for most of the cases since they were all negative for major potential diarrhoea-related pathogens.

Enteroviruses are pathogens that can invade and reproduce in intestinal cells and induce severe inflammation in human intestinal epithelial cells [17–19]. According to the pathogenic mechanisms proposed by Navaneethan and Giannella, enterovirus-related diarrhoea is initiated by inflammation involving activation of cytokines and inflammatory mediators [20]. EV71 and CVA6 were the two serotypes that claimed more than half of diarrhoea cases (15/28), the diarrhoea prevalences of different EV serotypes varied considerably: Echo 14 (100 %), CVA21 (100 %), Echo 2 (50.0 %), CVA9 (33.3 %), CVA10 (9.7 %), CVA4 (6.3 %), CVA6 (4.5 %), EV71 (2.3 %) and CVA16 (0.6 %). Although no particular serotype of enterovirus is especially associated with diarrhoea, previous studies have suggested that some serotypes of echovirus and coxsackievirus B are more likely to cause diarrhoea [10–12, 21, 22], which is similar to our results. Enteroviruses are generally not listed as potential pathogens for diarrhoea, which leads to limited information about enterovirus-related diarrhoea, and aetiological misdiagnosis. Therefore, physicians should consider enteroviruses as potential pathogens when treating diarrhoea, especially for cases admitted during times of seasonal enterovirus circulation. Moreover, more studies should be carried out to elucidate the discrepancies and clinical features of diarrhea caused by different serotypes of enterovirus due to the extreme lack of such knowledge.

Enterovirus-related diarrhoea did not did not exert substantial influence on the severity of HFMD/HA in patients who received appropriate and timely medical intervention. No difference was observed between the diarrhoea and no diarrhoea groups regarding duration of hospitalisation and prevalence of death, CNS involvement, severe CNS involvement, paralysis, altered consciousness, ARI and severe ARI. Although the diarrhoea group had significantly higher prevalence of MODS (10.7 vs 0.40 %), hepatic injury (14.3 vs 3.4 %), myocardial injury (21.4 vs 6.1 %) and convulsion (21.4 vs 7.2) than no diarrhoea group, all of these manifestations were short term and reversible with treatment. The limited influence of diarrhoea on severity of HFMD/HA was largely due to the following factors: the mild or moderate nature of most diarrhoea, without severe and long-term dehydration; appropriate and timely medical intervention; the inflammatory mechanism of enterovirus-related diarrhoea; and inability of enterovirus particles and proinflammatory cytokines to cross the blood–brain barrier in most conditions. Evasion and modulation of the gastrointestinal defence systems of the host are essential for pathogens causing infectious diarrhoea [20]. Thus, it is reasonable to assume that higher levels of cytokines and inflammatory mediators will be produced in HFMD/HA patients with than without diarrhoea. These higher levels can cause increased prevalence of hepatic injury, myocardial injury and MODS in HFMD/HA patients with diarrhoea. In our study, most cases of diarrhoea (82.1 %) presented without dehydration, and timely hospital treatment abolished dehydration when it did occur. Therefore, it was inflammation rather than haemodynamic disturbance caused by dehydration that was responsible for the differences between the diarrhoea and no diarrhoea groups. Because MODS was initiated by inflammation, there was no significant increase in mortality in the diarrhoea group when patients received timely medical intervention. The exceptionally high prevalence of convulsion in the diarrhoea group was largely because convulsion, unlike other neurological manifestations, can be induced in young children by mere dehydration or electrolyte imbalance. The absence of any substantial increase in prevalence of CNS involvement, severe CNS involvement and paralysis in HFMD/HA with diarrhoea was a relief for such patients.

Some limitations may exist because our study was a retrospective observation based on the enterovirus serotypes that were circulating in Guangdong province during 2009–2012. Moreover, the limited number of diarrhoea cases and other possible co-infections may also have biased the results. Intensive treatment administered during hospitalization also limited the natural progression of diarrhoea and even prevented its impact on the severity of HFMD/HA. Therefore, more studies should be carried out to elucidate the characteristics of enterovirus-related diarrhoea and their implications in HFMD/HA.

## Conclusions

From 2009 to 2012, patients with diarrhoea caused by circulating enteroviruses had mostly mild or moderate gastrointestinal symptoms. Timely intervention reduced disease severity and improved outcome in children with HFMD/HA. As a result of its mild severity and being initiated by inflammation, enterovirus-related diarrhoea did not exert a major influence on severity or outcome in HFMD/HA inpatients in Guangdong, China, during 2009–2012.

## Products

Pan-enteroviruses kit, EV71 kit, CA16 kit (Shanghai ZJ Bio-tech Co., LTD); ViiA™ 7 system (Life Technologies, USA); virus transport system (Copan Italia S.P.A, Italy); QIAamp Viral RNA Mini Kit system (Qiagen, Hilden, Germany).

## Abbreviations

ALT: alanine aminotransferase
ARI: acute respiratory infection
AST: aspartate aminotransferase
CK: creatine kinase
CK-MB: creatine kinase-MB
CNS: central nervous system
CVA: coxsackievirus A
Echo: echovirus
HFMD/HA: hand, foot and mouth disease/herpangina
MODS: multi-organ dysfunction syndrome
RT-PCR: reverse transcriptase polymerase chain reaction
UTR: untranslated region

## References

[1] National statutory report of incidence and death statistics of infectious disease. http://www.moh.gov.cn/publicfiles//business/htmlfiles/wsb/pyqxx/list.htm.

[2] Hsiung GD, Wang JR (2000). Enterovirus infections with special reference to enterovirus 71. J Microbiol Immunol Infect.

[3] Stalkup JR, Chilukuri S (2002). Enterovirus infections: a review of clinical presentation, diagnosis, and treatment. Dermatol Clin.

[4] Distribution of causes of death among children aged < 5 years. http://apps.who.int/gho/data/view.main.CMDRWORLD?lang=en.

[5] Nix WA, Oberste MS, Pallansch MA (2006). Sensitive, seminested PCR amplification of VP1 sequences for direct identification of all enterovirus serotypes from original clinical specimens. J Clin Microbiol.

[6] Ge S, Yan Q, He S (2013). Specific primer amplification of the VP1 region directed by 5’ UTR sequence analysis: enterovirus testing and identification in clinical samples from hand-foot-and-mouth disease patients. J Virol Methods.

[7] Ruuska T, Vesikari T (1990). Rotavirus disease in Finnish children: use of numerical scores for clinical severity of diarrhoeal episodes. Scand J Infect Dis.

[8] Chaimongkol N, Khamrin P, Suantai B (2012). A wide variety of diarrhea viruses circulating in pediatric patients in Thailand. Clin Lab.

[9] Maslin J, Kohli E, Leveque N (2007). Characterisation of viral agents with potential to cause diarrhea in Djibouti. Med Trop (Mars).

[10] Rao DC, Ananda Babu M, Raghavendra A (2013). Non-polio enteroviruses and their association with acute diarrhea in children in India. Infect Genet Evol.

[11] Abe O, Kimura H, Minakami H (2000). Outbreak of gastroenteritis caused by echovirus type 6 in an orphanage in Japan. J Infect.

[12] Oishi I, Maeda A, Otsu K (1979). An outbreak of diarrhea in school children associated with Coxsackie virus B-3 infection. Biken J.

[13] Li CS, Chan PK, Tang JW (2009). Prevalence of diarrhea viruses in hospitalized children in Hong Kong in 2008. J Med Virol.

[14] Zeng M, Chen J, Gong ST (2010). Epidemiological surveillance of norovirus and rotavirus diarrhea among outpatient children in five metropolitan cities. Zhonghua Er Ke Za Zhi.

[15] Lee MK, Chan PK, Ho II, Lai WM (2013). Enterovirus infection among patients admitted to hospital in Hong Kong in 2010: epidemiology, clinical characteristics, and importance of molecular diagnosis. J Med Virol.

[16] Li DD, Li H, Guo RN (2010). Pathogens of high incidence of other infectious diarrhea in Guangdong Province, from November 2008 to January 2009. Bing Du Xue Bao.

[17] Xu J, Yang Y, Wang C, Jiang B (2009). Rotavirus and coxsackievirus infection activated different profiles of toll-like receptors and chemokines in intestinal epithelial cells. Inflamm Res.

[18] Zhang YC, Jiang SW, Gu WZ (2012). Clinicopathologic features and molecular analysis of enterovirus 71 infection: report of an autopsy case from the epidemic of hand, foot and mouth disease in China. Pathol Int.

[19] Chi C, Sun Q, Wang S (2013). Robust antiviral responses to enterovirus 71 infection in human intestinalepithelial cells. Virus Res.

[20] Navaneethan U, Giannella RA (2008). Mechanisms of infectious diarrhea. Nat Clin Pract Gastroenterol Hepatol.

[21] Carvalho RP, Kirchner E, Moura RA, Murahovschi J (1977). Acute gastroenteritis associated with coxsackie B-6 virus in infants and children, in Sao Paulo, Brazil. Rev Inst Med Trop Sao Paulo.

[22] Tai FH, Wang HC (1973). Acute gastroenteritis associated with echovirus type 1. Southeast Asian J Trop Med Public Health.

